# Cool-edge populations of the kelp *Ecklonia radiata* under global ocean change scenarios: strong sensitivity to ocean warming but little effect of ocean acidification

**DOI:** 10.1098/rspb.2023.2253

**Published:** 2024-01-17

**Authors:** Damon Britton, Cayne Layton, Craig N. Mundy, Elizabeth A. Brewer, Juan Diego Gaitán-Espitia, John Beardall, John A. Raven, Catriona L. Hurd

**Affiliations:** ^1^ Institute for Marine and Antarctic Studies, University of Tasmania, 20 Castray Esplanade, Battery Point, Hobart, Tasmania 7004, Australia; ^2^ CSIRO Oceans and Atmosphere, Hobart, Tasmania 7000, Australia; ^3^ School of Biological Sciences and the SWIRE Institute of Marine Sciences, The University of Hong-Kong, Hong Kong, People's Republic of China; ^4^ School of Biological Sciences, Monash University, Clayton, Victoria 3800, Australia; ^5^ Division of Plant Science, University of Dundee at the James Hutton Institute, Invergowrie, Dundee DD2 5DA, UK; ^6^ School of Biological Sciences, University of Western Australia, 35 Stirling Highway, Crawley, Western Australia 6009, Australia; ^7^ Climate Change Cluster, University of Technology, Sydney, Ultimo, New South Wales 2007, Australia

**Keywords:** physiology, phenotypic plasticity, ocean warming, ocean acidification, thermal performance curves, multiple drivers

## Abstract

Kelp forests are threatened by ocean warming, yet effects of co-occurring drivers such as CO_2_ are rarely considered when predicting their performance in the future. In Australia, the kelp *Ecklonia radiata* forms extensive forests across seawater temperatures of approximately 7–26°C. Cool-edge populations are typically considered more thermally tolerant than their warm-edge counterparts but this ignores the possibility of local adaptation. Moreover, it is unknown whether elevated CO_2_ can mitigate negative effects of warming. To identify whether elevated CO_2_ could improve thermal performance of a cool-edge population of *E. radiata*, we constructed thermal performance curves for growth and photosynthesis, under both current and elevated CO_2_ (approx. 400 and 1000 µatm). We then modelled annual performance under warming scenarios to highlight thermal susceptibility. Elevated CO_2_ had minimal effect on growth but increased photosynthesis around the thermal optimum. Thermal optima were approximately 16°C for growth and approximately 18°C for photosynthesis, and modelled performance indicated cool-edge populations may be vulnerable in the future. Our findings demonstrate that elevated CO_2_ is unlikely to offset negative effects of ocean warming on the kelp *E. radiata* and highlight the potential susceptibility of cool-edge populations to ocean warming.

## 1. Introduction

Anthropogenic climate change is having unprecedented effects on the oceans, with rising temperatures and elevated CO_2_ concentrations having adverse impacts on nearly all marine ecosystems [1]. Of particular concern, is declines in a range of habitat forming species such as kelps (order Laminariales, [2,3]), which support diverse and productive ecosystems in temperate and subpolar regions globally [4,5]. Substantial research effort has been undertaken in the last 10–15 years to identify kelp species and communities that are vulnerable to global ocean change [3,6,7]. However, there remain significant gaps in our understanding of the combined effect of multiple co-occurring drivers such as temperature and CO_2_ [8,9]. Identifying key interactions between major components of global ocean change such as elevated temperature and dissolved CO_2_ and identifying the threshold at which a driver such as temperature becomes a stressor is urgently required to improve our ability to accurately predict changes and identify vulnerable species and communities.

Since 1850, the surface of the ocean has warmed by approximately 1°C, with a further 2–3°C increase projected by the end of the century depending on which emissions scenario is realized (ocean warming, [1]). Many kelps are highly sensitive to thermal stress as evidenced by the declines and local extinctions associated with ocean warming [3,6,10] and extreme and acute warming events (i.e. marine heatwaves; [11–13]). Further losses of kelp have been projected throughout the twenty-first century [14]. For example, modelling work by Martínez *et al*. [14] suggests up to 70–100% declines in the distrubution of multiple Australian kelps and prominent fuciods by 2100. However, much research has focused on populations at the warm edge of a species' range (e.g. [12,15]), which are already experiencing thermal stress. Often overlooked, however, are populations from cooler edges of species' ranges, which are thought to be more tolerant to ocean warming as they exist below the thermal optima of the species. As such, our knowledge of intraspecific variation in thermal tolerance and the wider impact of ocean warming across species ranges remains limited [16,17]. This is problematic as, by assuming warm-edge populations are more susceptible, we may consider that range contractions under climate change will be linear and ignore the susceptibility of populations adapted to cooler waters [18]. There is growing evidence that the thermal tolerances of populations from different thermal regimes differ due to local adaptation for kelps [18,19], corals [20,21] and invertebrates [22,23]. Identifying whether these cool-edge populations are equally susceptible is therefore key to providing a better understanding of how a species as a whole will respond to ocean warming.

One way to improve our understanding of kelps' vulnerability to ocean warming is to generate population-level thermal performance curves (TPCs, [24,25]). TPCs describe the relationship between a physiological process (e.g. photosynthesis or growth) over a wide range of temperatures. Physiological performance (referred to hereafter as performance unless stated otherwise), as a function of temperature, can then also be used to model a species response to changing temperatures. This can lead to an improved understanding of a species performance over yearly cycles, identify potential sub-lethal effects, and periods of highest susceptibility to extreme warming events. However, performance is not only influenced by temperature but also by the interactions of other environmental drivers, which are often overlooked in projections of how kelps will respond to climate change [14]. Understanding the interactive effects of co-occurring drivers, such as dissolved CO_2_ and temperature, is urgently required, as responses across taxa can be varied. For example, both algal turfs and seagrass display enhanced thermal tolerance under elevated CO_2_ [26,27], whereas the two drivers can act synergistically to reduce performance in crustaceans [28]. Identifying the nature of these interactions for foundation species such as kelps is critical for improving the reliability and accuracy of predictions of how species will respond to global ocean change.

The sustained absorption of atmospheric CO_2_ has led to an increase in the dissolved CO_2_ concentrations of the surface ocean of approximately 70% and subsequent changes to the seawater carbonate system (ocean acidification, [1]). By 2100, dissolved CO_2_ levels are projected to be approximately 150–200% higher than in 1850 depending on which emissions scenario is realized [1]. As CO_2_ is required for photosynthesis, elevated CO_2_ concentrations are likely to have vastly different effects to that of ocean warming [29,30]. It is unlikely that kelps are carbon limited under today's CO_2_ concentrations, as they possess a carbon dioxide concentrating mechanism (CCM). CCMs increase the kelp's affinity for inorganic carbon by increasing CO_2_ concentrations at the active site of Rubisco, ensuring photosynthesis is essentially CO_2_-saturated under present-day concentrations [31,32]. As such, the idea that CO_2_ will have a ‘fertilizer effect' and directly increase productivity of kelps is unlikely [30,33]. However, if kelps can downregulate the CCM to rely more on diffusive uptake of CO_2_ as an inorganic carbon source, then this may endow an energetic saving via a reduced need to operate CCMs, which could be used to enhance growth or acclimatize to elevated temperatures [34]. Energetic savings arising from CCM downregulation has been proposed as a way in which the habitat forming fucoid *Phyllospora comosa* is able to tolerate elevated temperatures [34]. The proposed mechanism suggests that energetic savings arising from CCM downregulation are used to alter cellular membrane fatty acid composition, which in turn reduce the negative effects of increased membrane fluidity at high temperatures [34]. A similar mechanism occurs in response to elevated inorganic nitrogen conditions in the kelp *Macrocystis pyrifera* (giant kelp), where adjustments in fatty acid composition increases thermal optima [35,36]. Elevated CO_2_ has been shown to enhance performance at elevated temperatures in algal turfs [26], seagrass [27] and terrestrial plants [37], suggesting this may be a widespread phenomenon; however, it requires further investigation in kelps.

In Australia, kelp forests form the foundation of the Great Southern Reef, a continental-scale temperate rocky reef system which is a global hotspot for marine biodiversity and endemism [19]. The region is also an ocean warming hotspot, with both the waters of southeast and southwest of Australia warming at rates of three to four times the global average [38]. This warming has already been linked to approximately 95% declines in the canopy cover of *M. pyrifera* forests in Tasmania, southeastern Australia, since the 1970s [10,39]. The most abundant and widespread kelp across the broader Great Southern Reef is *Ecklonia radiata* (C.Agardh) J.Agardh*,* which has a widespread distribution from subtropical latitudes (approx. 27° N) on both east and west coasts of Australia, to the cold temperature regions in southern Tasmania (approx. 44° S, [40]). The species can survive temperatures as high as 28°C, and the optimum temperature for net photosynthesis of *E. radiata* sporophytes in Western Australia is 24°C [15]. Moreover, the gametophyte life stage displays evidence of acclimatization or adaptation to local thermal regimes, with higher thermal optima positively correlated with higher *in situ* temperatures [41]. Recent modelling work by Young *et al*. [42] demonstrated the potential for declines in *E. radiata* cover in cool-edge populations of the southeastern Australian state of Victoria, with one of the main drivers being increased sea surface temperatures. However, there is little understanding of the thermal tolerance of *E. radiata* sporophytes at the physiological level from populations at the cool edge of its distribution, and no studies have assessed whether thermal performance is likely to change with the elevated levels of CO_2_ projected under global ocean change. Accordingly, the aims of this study were threefold: (i) identify the thermal optima for growth and photosynthesis for cool-edge populations of *E. radiata* in Australia, (ii) examine whether thermal performance of growth and photosynthesis changes under elevated CO_2_, and (iii) use these modelled relationships to identify timepoints in which performance of this cool-edge population of *E. radiata* will decline under global ocean change scenarios. The results of this study highlight a previously unrecognized susceptibility of cool-edge populations of *E. radiata* to ocean warming, which is unlikely to be mitigated by ocean acidification.

## 2. Material and methods

### (a) Collection and acclimatization

Approximately 100 individual juvenile *E. radiata* sporophytes of approximately 10 cm length were collected on 2 February 2021 from Coal Point, Tasmania (43.33430° S, 147.32493° E) from 6–8 m depth by divers on SCUBA. Individuals were placed in an insulated container in darkness in seawater following collection and transported back to the laboratory approximately 2 h away. Once in the laboratory, all individuals were acclimatized to laboratory conditions by placement in a common 40 l container containing filtered seawater (0.2 µm pore size) in a temperature-controlled room set at 16°C, this was similar to the field site which had an average sea surface temperature (SST) in preceding 14 days of 17.87°C [43]. Lighting was provided by dimmable LED lights (Zeus-70, Ledzeal, Hong Kong), that mimicked the colour spectrum present at approximately 8 m water depth on a 12 : 12 light dark cycle. Mean irradiance was 70 µmol m^−2^ s^−1^; however, this varied over the day to mimic a natural light cycle, increasing linearly from 0 to 130 µmol m^−2^ s^−^^1^ between 06.00 and 11.00, and then maintained at 0–130 µmol m^−2^ s^−1^ until 13.00 when it then decreased linearly again back to 0 µmol m^−2^ s^−1^ at 18.00. Individuals were kept under these conditions for 72 h.

### (b) Experimental culture conditions

Following laboratory acclimatization, 80 individuals were haphazardly allocated to one of eight 15 l containers, filled with filtered and aerated seawater at ambient CO_2_ levels. The temperature of each container was then increased/decreased to the experimental temperature (6, 9, 12, 16, 20, 23, 26 and 29°C) at a rate of 2°C per day. When each bath reached its target experimental temperature (0–6.5 days depending on the treatment), those individuals were each attached to a small pebble (approx. 30 mm ∅) by their holdfast using a rubber band, and haphazardly allocated to one of 10 2 l experimental chambers. Within each temperature treatment, half of the chambers were maintained at ambient pCO_2_ (target 420 µatm) and the other half at the future pCO_2_ (target 1000 µatm, simulating the upper projections of representative concentration pathway (RCP) 8.5, [1]) level giving a total of five replicates (*n* = 5) for each of the 16 unique combinations of temperature and CO_2_ (*N* = 80). For each treatment, the experiment was considered to have begun at this point, therefore the starting date for each treatment was staggered depending on the time to reach the target temperature. Each treatment ended after 21 days at the experimental temperature.

The experiment took place within the same temperature-controlled room as the laboratory acclimatization, which was set at 16°C. Temperature treatments were maintained using water baths that were modified using either chiller units (6, 9, 12°C; RC1, Ratek Instruments and C2G, Grant Instruments) or heaters (20, 23, 26 and 29°C; T100, Grant Instruments and Aqua One IPX8, Kongs Australia) all via independent temperature-controller units (T100, Grant Instruments and Temperature Controller 7028/3, Tunze). The 16°C water bath remained at the ambient temperature of the temperature-controlled room. Temperatures in each bath were monitored and recorded using temperature loggers (HOBO Pendant MX Temp, Onset Computer Corporation). Water in each experimental chamber was replaced every 3–4 days for the duration of the acclimatization and experiment to allow replenishment of seawater nutrient concentrations depleted by the kelp. Water samples for determination of nitrate and ammonium concentrations were taken in a subset of chambers just prior to the final water change of the experiment. Samples were filtered to 0.7 µm (GF/F, Whatman) and immediately frozen at −20°C in 12 ml polyethylene vials. Samples were subsequently thawed, and ammonium and nitrate concentrations were determined using a QuickChem 8000 Automated Ion Analyser (LaChat Instruments).

The two pCO_2_ treatments were maintained by constant bubbling of each chamber with either air (for ambient pCO_2_) or an elevated CO_2_/air mix (for future pCO_2_). Filtered air was provided by outlets in the temperature control room sourced within the building and CO_2_ provided by a single CO_2_ cylinder (CO_2_ food grade, BOC Gas and Gear). Elevated CO_2_/air mixes were controlled by adjusting the voltage on mass flow controllers (FMA5418A and FMA5402A, Omega Engineering). These voltages were maintained for the duration of the experiment to ensure constant delivery of the same pCO_2_ to the enriched chambers. CO_2_ levels were not increased slowly, as for temperature, as pilot studies and previous experiments indicated no adverse effects of instantaneously adding elevated CO_2_. All experimental chambers had a small outlet to allow the bubbled gas to escape from the otherwise sealed container. To monitor the concentration of dissolved inorganic carbon (DIC), pH was monitored daily using a spectrophotometric pH system [46] in randomized chambers from each of the 16 unique combinations of temperature and pCO_2_ daily until the end of the first treatment on 25 February 2021. Samples of seawater for determination of total alkalinity were taken from a random chamber within each of the 16 unique experimental combinations (two samples per combination) on 19 February 2021. Samples (approx. 60 ml) were filtered to 0.2 µm and immediately poisoned with HgCl_2_ (0.02% v/v) until later analysis. Total alkalinity was analysed using a total alkalinity titrator (862 Compact Titrosampler, Metrohm) using best practice methods [47]. DIC and pCO_2_ were calculated in CO2SYS [48] using the constants of Mehrbach *et al*. [49] refit by Lueker *et al*. [50] and the known A_T_, pH, temperature and salinity (measured with an Orion Versa Star Advanced Electrochemistry Meter, ThermoFisher Scientific) of the seawater.

### (c) Biotic responses

#### (i) Growth (linear extension) and change in wet weight (%)

Growth was measured to provide information on the capacity of the kelp to increase biomass, and change in wet weight provided a holistic measure of growth (increase in biomass) minus tissue loss (erosion). To measure rates of linear extension, a single 5 mm diameter hole was punched in the blade of each individual above the meristem prior to photographing on day 1 [51]. Photographs were again taken at the end of the experiment on day 21 and the distance of the hole from the base of the blade was calculated using the software Fiji [52]. Linear extension was calculated as the length of the hole from the base of blade on day 21 minus length of hole from base of blade on day 1 and is expressed as mm day^−1^. Wet weight was measured by weighing each individual after they were gently patted dry on day 1 and day 21. Change in wet weight was calculated as the percentage change from the initial weight on day 1.

#### (ii) Net photosynthesis

Oxygen evolution in the light (net photosynthesis, a key indicator of performance) was measured on day 21 (i.e. after each individual had been exposed to experimental treatments for three weeks) for each of the five replicates per temperature and CO_2_ combination. All incubations were conducted at the experimental irradiance and CO_2_, and temperature was maintained at the appropriate levels for each treatment. Individuals were placed in sealed 260 ml glass chambers on an orbital shaker (OM7 Large Orbital Shaker, Ratek Instruments). Oxygen concentrations were measured at time = 0 and time = 3 h with a portable oxygen meter (Fibox 4, PreSens), coupled with a non-invasive oxygen sensor in each culture chamber (Oxygen Sensor Spot SP-PSt3-NAU, PreSens). Net photosynthesis is expressed as µmol O_2_ g^−1^ h^−1^ wet weight. Values were corrected using the average change in oxygen concentration in three ‘blank' control chambers containing no algae for each CO_2_ and temperature combination.

#### (iii) Stable C isotopes and C and N content

Stable isotope ratios were measured to provide evidence of inorganic carbon uptake strategies and C and N content were measured to provide indications on the nutrient status of the kelp. Samples for determination of *δ*^13^C isotopic ratios and carbon and nitrogen content were destructively sampled and frozen at –20°C until all treatments had been measured. Following this, all samples were freeze dried (FreezeZone 4.5, Labconco) and kept at –20°C until later analysis. *δ*^13^C isotopic ratios and carbon and nitrogen content were determined by weighing approximately 5 mg of dried tissue into tin cups (Sercon, UK) and analysed using an elemental analyser (NA1500, Fisons Instruments) coupled to an isotope ratio mass spectrometer (Delta V Plus, ThermoFisher Scientific) via a Universal Continuous Flow Interface (Conflo IV, ThermoFisher Scientific). Combustion and reduction were achieved at 1020°C and 650°C, respectively. Isotope values were normalized to the Vienna Pee Dee Belemnite scale via a three-point calibration using certified reference material and both precision and accuracy were ±0.1% (1 s.d.).

### (d) Statistical analysis

#### (i) Model fitting and selection

All analyses were conducted in the statistical software program R v. 3.6.1 [53]. As multiple functions can be used to model TPCs, we fitted nine models that are commonly used for each response variable (growth, net photosynthesis and change in wet weight) at each CO_2_ level. Briefly, each model followed a pattern typical of TPCs where performance increases from low temperatures until an optimum, after which performance rapidly declines. All models and their outputs and can be seen in electronic supplementary material, table S1 and further reading on each of the nine models can be found in Padfield *et al*. [54]—electronic supplementary material. The most appropriate model for each response and CO_2_ level was chosen by firstly excluding all models that had a root mean square error (RMSE) greater than 2% of the best fitting model; secondly, we excluded models that were non-sensical (e.g. those that didn't follow a known pattern of thermal performance or included negative values where these were not possible such as linear extension), and thirdly if more than one model was deemed appropriate, the model with the most useful biological parameters was chosen with priority given to models that explicitly included thermal optima and maximum rate followed by critical thermal maximum. This approach was chosen as it balanced goodness of fit and useful biological information [55]. All models were fitted using nonlinear least squares using the package *rTPC* [56] or *stat*s [53]. The parameters of each model were extracted from the best fit model using the *rTPC* package [54] with 95% confidence intervals around the model fits and parameter estimates derived by bootstrapping using the *car* package [57]. A quadratic function was used to model the effect of temperature on stable C isotopes for both current and future CO_2_ levels. In the highest temperature treatment, mortality occurred in four individuals from the current CO_2_ level and two from the future CO_2_ level. Individuals were considered dead if they had both net photosynthetic rates of below zero and substantial loss of tissue. These individuals were excluded from the net photosynthesis models to avoid them having excessive influence on model fits. Tissue samples from the dead individuals were still included in measurements of stable C isotopes and C and N content, except for one in the current CO_2_ level, which did not have enough remaining tissue for analyses due to tissue loss. A measurement error also resulted in the loss of one sample from the 20°C in each of the current and future CO_2_ levels for net photosynthesis.

#### (ii) Projections of thermal performance

To illustrate how thermal performance may change under different warming scenarios, we modelled annual thermal performance for growth, net photosynthesis and change in wet weight under three scenarios: (i) current: average daily SST between 2002 and 2020 at the collection site [43], (ii) future: average daily SST between 2002 and 2020 + 3°C, and (iii) future extreme: average daily SST between 2002 and 2020 + 4.5°. The two future scenarios were chosen to represent the upper end of projections of SST under RCP 4.5 and RCP 8.5 in 2090 for this region [58]. To model performance, we populated the models chosen to describe the experimental TPC responses for each response variable (i.e. those in table 1) for the SST conditions described above. Model fits under current CO_2_ were used for the current scenario and model fits under elevated CO_2_ were used for both future scenarios. Model projections were made by using the *predFit* function in the *investr* package [59] with 95% confidence intervals calculated by bootstrapping in the *car* package [57]. All projections display change in performance relative to the current scenario and are expressed as percentage change in performance. As such, all projections display how thermal performance will vary relative to the present day and are not intended to be seen as projections of realized performance which will be influenced by a multitude of biotic and abiotic factors.

**Table 1.** Model parameters for each model fitted to thermal performance curves under both current and future CO_2_. *T*_opt_ = thermal optimum and *R*_max_ = maximum rate at thermal optimum. Values are model estimates of each parameter with 95% confidence intervals in parentheses.

**response**	**CO_2_** **level**	**model**	*T*_opt_	*R*_max_
growth (linear extension)	current	O'Neill *et al*. [44]	16.05 (14.68–17. 30)	1.01 (0.86–1.17)
	future	O'Neill *et al*. [44]	15.72 (14.94–16.57)	1.06 (0.94–1.27)
net photosynthesis	current	O'Neill *et al*. [44]	16.67 (5.57–23.72)	4.65 (3.72–5.82)
	future	O'Neill *et al*. [44]	18.44 (16.30–21.34)	7.48 (6.47–8.54)
change in weight %	current	Yan and Hunt [45]	16.60 (15.39–18.06)	43.04 (37.84–48.72)
	future	O'Neill *et al*. [44]	14.80 (13.83–15.84)	50.95 (38.14–64.96)

## 3. Results

### (a) Experimental culture conditions

Temperatures were maintained close to target values and ranged from a mean of 5.57°C in the lowest temperature treatment to 28.84°C in the highest temperature treatment. pCO_2_ values were on average 421.98 ± 65.295 µatm (s.e.) in the current CO_2_ treatments and 992.35 ± 151.90 (s.e.) in the future CO_2_ treatments. There was some variation in pCO_2_ levels within both current and future treatments ranging from 380 to 472 µatm in the current treatment and 840 to 1156 µatm in the future treatment, presumably due to the metabolic activity of the kelp altering inorganic carbon concentrations. Electronic supplementary material, table S2 shows the specific means and standard errors for temperature and pCO_2_ for each of the 16 treatment combinations. Nitrate and ammonium concentrations measured just prior to a water change were 1.28 µM ± 0.22 and 0.54 µM ± 0.42 respectively (means and s.d., *n* = 47).

### (b) Biotic responses

#### (i) Growth (linear extension), net photosynthesis and change in wet weight (%)

**Figure 1.**
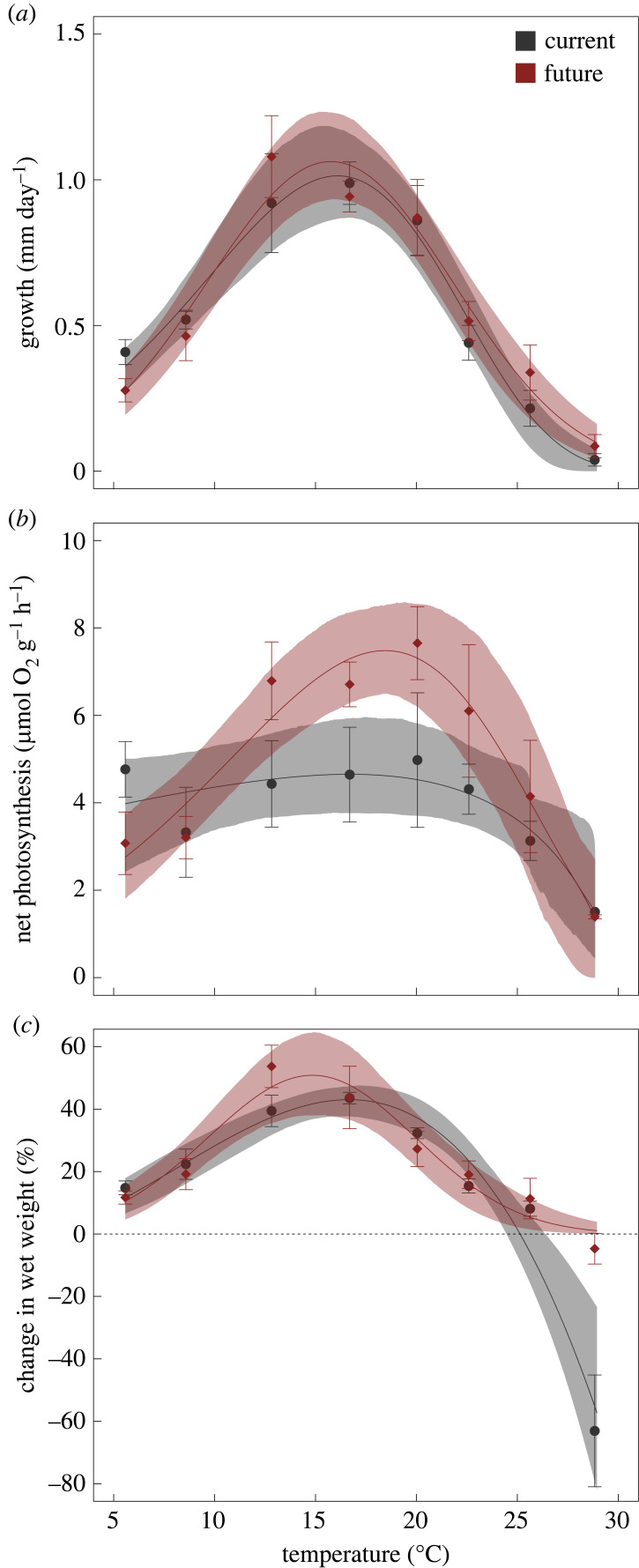
Thermal response curves of *E. radiata* individuals cultured at 5.87–28.84°C under current (422 µatm, grey lines) and future (992 µatm, red lines) pCO_2_ conditions. Shaded areas refer to 95% confidence intervals of model fits. Grey circles refer to mean values under current pCO_2_ and red diamonds refer to mean values under future pCO_2_, error bars are standard error, *n* = 1–5 for each temperature and pCO_2_ combination. (*a*) growth (mm d^−1^), (*b*) net photosynthesis (µmol O_2_ l^−1^ g^−1^ h^−1^), (*c*) change in wet weight (%).

Linear extension, net photosynthesis and change in wet weight all followed patterns typical of TPCs with rates increasing from low temperatures until an optimum, followed by a decline at temperatures beyond the optimum (figure 1). There was minimal effect of CO_2_ on linear extension rates with near identical curves fitting both data and thermal optimum and maximum growth rates being similar, with overlapping confidence intervals (table 1). For net photosynthesis, thermal optima were similar under both current and future CO_2_ levels with similar means (current = 16.86, future = 18.01) and overlapping confidence intervals (current = 5.57–20.43, future = 16.50–19.56, table 1). However, the maximum rate of net photosynthesis at these optima differed (mean current = 4.67 µmol O_2_ l^−1^ g^−1^ h^−1^, mean future = 7.28 µmol O_2_ l^−1^ g^−1^ h^−1^) and 95% confidence intervals did not overlap (current = 3.80–5.85 µmol O_2_ l^−1^ g^−1^ h^−1^, future = 6.49–8.56 µmol O_2_ l^−1^ g^−1^ h^−1^, table 1). Changes in wet weight were similar between current and future CO_2_ levels for both thermal optima and maximum rate of change (overlapping confidence intervals, table 1). However, there was substantially greater tissue loss at 28.84°C in the current CO_2_ level treatment as evidenced by percentage change in weight (means ± 95% confidence intervals: current = −63.06 ± 49.68, future = −4.71 ± 13.63).

#### (ii) Stable C isotopes and C : N ratios

Carbon stable isotope values followed a pattern of being reduced at low temperatures, increasing until approximately 16°C and then declining until the highest temperature (electronic supplementary material, figure S1). This pattern was well described by a quadratic function for both CO_2_ scenarios, and model fits were similar for both current and future CO_2_ levels with overlapping confidence intervals. Means ± s.e. for % N, % C and C : N at each temperature and CO_2_ level are shown in table 2.

**Table 2.** Means and standard error for % C, % N and C : N ratios in *E. radiata* cultured at each temperature under both current and future pCO_2_ levels.

**CO_2_level**	**Temperature °C**	**% C**	**s.e.**	**% N**	**s.e.**	**C : N**	**s.e.**
**current**	5.57	33.3	0.45	1.1	0.03	30.5	1.0
	8.58	35.9	1.07	1.3	0.18	29.5	3.1
	12.82	33.3	0.54	1.0	0.07	31.8	2.0
	16.69	35.1	0.45	0.9	0.02	36.6	0.7
	20.04	32.2	0.64	0.9	0.02	32.7	0.9
	22.59	28.9	0.61	0.9	0.02	29.7	1.0
	25.63	34.2	0.57	1.0	0.04	32.2	1.0
	28.84	28.7	0.76	1.4	0.11	20.8	2.5
						
**future**	5.57	34.4	0.56	1.1	0.04	29.5	1.3
	8.58	36.5	0.32	1.0	0.02	36.2	1.0
	12.82	33.5	0.30	1.0	0.02	33.7	0.7
	16.69	34.3	0.42	1.0	0.02	36.2	0.4
	20.04	33.0	0.36	1.0	0.04	34.9	1.4
	22.59	29.9	0.50	0.9	0.04	31.3	1.1
	25.63	35.1	0.76	1.0	0.03	34.5	1.7
	28.84	27.6	0.99	1.4	0.10	19.7	1.9

### (c) Projections

**Figure 2.**
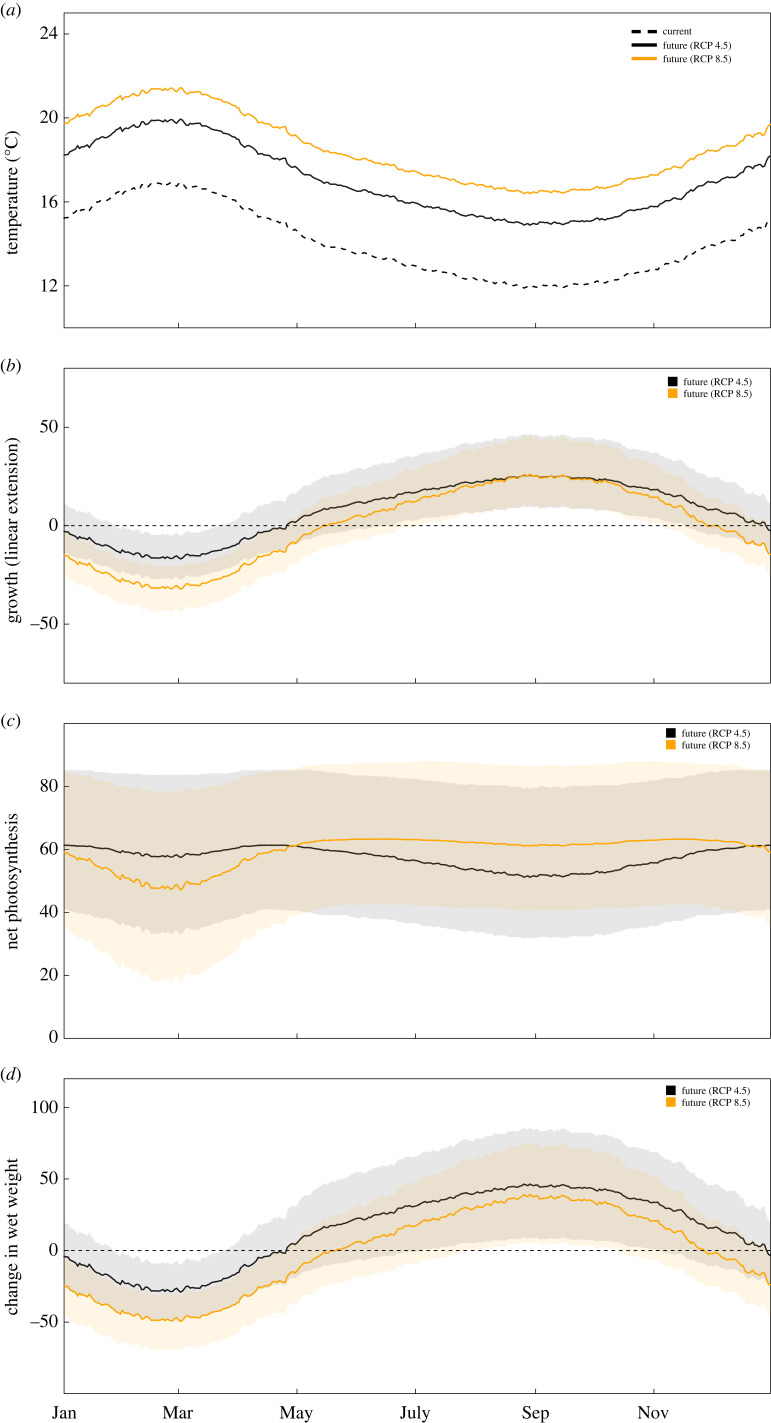
Modelled annual performance of *E. radiata* over the course of a year based on average SST at the collection site and additional increases in temperature to simulate global ocean change scenarios (note that this is a Southern Hemisphere site, thus January is summer). Performance is displayed as % increase or decrease relative to current conditions. (*a*) daily SST average in each scenario at collection site, (*b*) growth (linear extension), (*c*) net photosynthesis, (*d*) change in wet weight. Shaded areas refer to 95% confidence intervals of model fits.

Modelled annual thermal performance varied considerably in both warming scenarios (figure 2). All percentage changes are relative to thermal performance under present thermal regimes using the elevated CO_2_ models. When imposing an additional 3°C on baseline temperatures to simulate future ocean conditions the model indicated an increase in growth rates during late winter and early spring (August–October, peak 26% increase) and a decrease during late summer and early autumn (February–April, peak 17% decrease). A similar pattern was observed for change in weight, with a peak increase of 46% in spring and a peak decrease of 29% early autumn. By contrast, net photosynthetic rates increased at all stages of the year (51–61% increase). Imposing a 4.5°C increase on current baseline temperatures to simulate an extreme warming scenario indicated that in winter and early spring growth rates would increase (peak 26%) and decrease in late summer and early autumn (peak 33%). Photosynthetic rates increased throughout the year (47–63% increase), while weight change increased in winter and spring (peak 39% increase) and decreased by in late summer and autumn (peak 49% decrease).

## 4. Discussion

We measured thermal performance of juveniles of the habitat forming kelp *E. radiata* from a population at the cool edge of its distribution under ambient and elevated CO_2_, to identify thermal optima and tolerance and whether these were influenced by elevated CO_2_. Optimum temperatures for growth and photosynthesis were unaffected by CO_2_ and were substantially lower (approx. 6°C) than reported for sporophytes of *E. radiata* at the warm edge of its distribution [15]. Maximum growth rates were unaffected by CO_2_; however, maximum net photosynthetic rates were 59% higher under elevated CO_2_. Using modelled thermal performance under both current and future CO_2_ conditions and historical SST data, we projected performance under future global ocean change scenarios. These model projections identified that this cool-edge population of *E. radiata* in Australia is susceptible to ocean warming and marine heatwaves in late summer and early autumn. This highlights the importance of understanding and mitigating additional stressors over these periods of risk and has implications for reproductive phenology, productivity, and more broadly for the seasonality of restoration and aquaculture.

### (a) CO_2_: effect on growth and photosynthesis across temperatures

Elevated CO_2_ had varying effects on the performance of *E. radiata* juveniles across temperatures. Growth rates were unaffected, except for tissue loss being mitigated under elevated CO_2_ at temperatures above 26°C. The lack of an increase in thermal tolerance was unexpected as elevated CO_2_ has been shown to increase thermal performance in the fucoid *P. comosa* [34], as have elevated concentrations of inorganic nitrogen in the kelp *M. pyrifera* [35]. In contrast to growth, rates of net photosynthesis increased, but only around the optimum temperatures (i.e. changes in the *Y*-axis of the TPC). The decoupling of the growth and photosynthetic response is not surprising as increases in photosynthetic rate due to elevated CO_2_ are not always paralleled by differences in growth rate [60]. However, the mechanisms driving the elevated photosynthetic rates around thermal optimum are unclear. Carbon isotope discrimination values suggested the presence of a CCM in *E. radiata* [32,61], yet we found no evidence for the downregulation of the CCMs as previously observed in *E. radiata* [62]. It is possible that elevated CO_2_ stimulated an increase in Rubisco content as seen in green algae [63]. However, stress associated with extreme temperatures was probably limiting photosynthetic rates at the extremes [64], meaning *E. radiata* was unable to benefit from the additional CO_2_. Alternatively, the specificity factor of Rubisco for CO_2_/O_2_ generally decreases with elevated temperatures [65], which could have limited net photosynthesis above the optima due to increases in photorespiration. While at lower temperatures, rates of chemical reactions are limited by temperature and additional Rubisco would need to be synthesized to increase photosynthetic rates [66]. However, since we did not measure Rubisco kinetics or content, we are unable to identify specific mechanisms. Further research using molecular tools such as transcriptomics and gene expression would assist in elucidating some of these putative mechanisms.

### (b) Warming profiles and local adaptation

The thermal optimum for this cool-edge *E. radiata* population was substantially (6–8°C) lower than reported previously for the species from the warm edge of its distribution ([15], approx. 24°C). It is important to note that the study of Wernberg *et al*. [15] measured short-term (45 min) incubations, whereas our study was conducted over three weeks, making direct comparisons difficult. Nevertheless, this observed difference in thermal optima may indicate substantial acclimatization or adaptation of *E. radiata* populations to local temperature conditions [67,68], supporting previous findings of similar adaptation or acclimatization for the gametophyte life stage [41,69]. Moreover, the difference in optima is similar to the difference in annual mean SST between our site and those in Wernberg *et al*. [15] of approximately 6–8°C, further suggesting acclimatization or adaptation to local temperatures. This has implications for the cool-edge populations of *E. radiata* as they may be more susceptible to warming and marine heatwaves than was previously thought (e.g. [14]).

While it is unclear whether the differences we observed are due to acclimatization, adaptation or some combination of both, understanding these mechanisms is critical to assessing overall susceptibility to warming. If their thermal tolerance is related to a distinct genetic identity and adapted cluster of cool-edge populations, there may be limited capacity to respond and overcome the projected increases in ocean warming [68,70,71]. Alternatively, since thermal performance can be a plastic trait that responds to environmental conditions (particularly at juvenile life-history stages, [72,73]), cool-edge populations may be able to respond and acclimatize as the region continues to warm. Critically, however, both the magnitude of warming and genetic connectivity are likely to be key determinants of future adaptive responses along thermal gradients [68,74]. Connectivity between *E. radiata* populations varies across its range but can be reasonably high [75]; although whether it is sufficient to facilitate adaptation in this global ocean warming hotspot [38] remains to be tested. Reciprocal transplants or common garden experiments could help characterize and differentiate patterns of adaptive potential, while further population genetic work is also needed to better understand broader patterns of gene flow across the species' range.

### (c) Identifying susceptible periods, and implications

The modelled annual profiles of *E. radiata* performance suggest juveniles of *E. radiata* at this location are most susceptible to global ocean change in late summer and early autumn, with growth rates declining in all modelled warming scenarios. However, these scenarios did not consider the influence of other key seasonal drivers of kelp performance such as light intensity, daylength, nutrient concentrations and grazing rates (e.g. [76,77]), and thus should not be considered a measure of realized performance over the course of a year. Regardless, they are valuable in providing indications of during which time periods *E. radiata* is likely to be under thermal stress*.* Identifying these time periods is critically important given the highly seasonal demographic patterns of *E. radiata* and kelps in general [76,78]. For example, highest thermal stress occurs in late summer and early autumn, which are the peak periods of growth for adult and juvenile *E. radiata* [79,80] and coincide with peak spore production for *E. radiata* in Tasmania [76,81]. Thus, increased thermal stress during that period may have particularly significant impacts on *E. radiata* population dynamics and subtidal kelp communities, beyond reductions in the growth rates of individuals.

The modelled projections of performance also highlighted likely periods of elevated growth in spring because of temperatures becoming closer to optimum during this time. This has implications for the timing of restoration interventions or aquaculture operations [80,82,83], and for broader ecosystem productivity during spring blooms. Yet, it is unlikely that these forecast periods of elevated growth would offset the negative impacts that arise during the period of increased thermal stress, particularly if marine heatwave events, which are predicted to increase in intensity and frequency, impose further thermal stress [84,85]. For example, if a thermal stress is severe enough in late summer/early autumn, there may be few *E. radiata* remaining to benefit from improved springtime growth rates. Even in less extreme cases, pervasive sublethal effects during periods of high stress may shape broader population dynamics into a sort of annual crash-and-recovery pattern (e.g. [86]). Similar patterns seem increasingly evident in giant kelp (*M. pyrifera*) in Tasmania (C Layton 2023, personal observation, [10]), which has experienced dramatic declines despite local seawater temperatures being within a suitable range for the species throughout much of the year, although this is probably in part also driven by nutrient limitation [10,17]. The loss, or reduced performance, of canopy-forming adults of *E. radiata* during periods of increased stress may also impact the growth and survivorship of juvenile conspecifics and the overall resilience and recovery potential of the system [80]. Analysing whether and how population dynamics of *E. radiata* will be impacted by warming and marine heatwaves in the natural setting should be a priority of future research. Characterizing patterns of intraspecific variability at regional and individual levels should also help to identify populations that may be particularly susceptible or resilient to ocean change (e.g. [17,74]), even for cool-edge populations that may be at higher risk of thermal stress than previously thought.

## 5. Conclusion

Typically, warm-edge populations of organisms, living near their thermal maximum, are considered more threatened by warming and local extinctions and range contractions. By contrast, cool-edge populations may be expected to benefit as warming increases temperatures towards the thermal optima for the species. However, here we show that thermal optima for sporophytes from a cool-edge population of the habitat forming kelp *E. radiata* can be substantially lower than that of warm-edge conspecifics, probably due to acclimatization/adaptation to local temperature regimes. Furthermore, we show that their thermal tolerance and optima are unlikely to be enhanced under the forecast increases in CO_2_ that are occurring alongside warming. Indeed, projections of global ocean change scenarios indicate that this cool-edge population of *E. radiata* is likely to become increasingly impacted in a future ocean. Given the geographical isolation of cool-edge populations of *E. radiata* in Tasmania and the lack of a benefit of elevated CO_2_, these cool-edge populations may be more threatened by warming than previously recognized. It is likely that rapid acclimatization/adaptation of *E. radiata* populations at regional scales may be required to counteract the negative effects of warming and marine heatwave events.

## Data Availability

The data are provided in electronic supplementary material [87].
